# Association between the use of contact force-sensing catheters and cardiac tamponade in atrial fibrillation ablation

**DOI:** 10.1007/s10840-019-00516-z

**Published:** 2019-02-02

**Authors:** Nian Liu, Qianqian Zhao, Linling Li, Mengxia Zhang, Jiao Huang, Sheng Wei, Songnan Wen, Yanfei Ruan, Xueyuan Guo, Songnan Li, Chengxi Jiang, Ribo Tang, Caihua Sang, Deyong Long, Ronghui Yu, Rong Bai, Xin Du, Jianzeng Dong, Changsheng Ma

**Affiliations:** 1 Department of Cardiology, Beijing Anzhen Hospital, National Clinical Research Center for Cardiovascular Diseases, Capital Medical University, No. 2 Anzhen Road, Beijing, 100029 China; 2 Department of Epidemiology and Health Statistics, School of Public Health, Tongji Medical College, Huazhong University of Science and Technology, Wuhan, Hubei China

**Keywords:** Atrial fibrillation, Radiofrequency ablation, Contact force–sensing catheters, Cardiac tamponade

## Abstract

**Introduction:**

Cardiac tamponade is a rare but life-threatening complication during atrial fibrillation (AF) catheter ablation. Contact force (CF)–sensing catheters improve ablation effectiveness. However, the impact of the application of CF-sensing catheters on the occurrence of cardiac tamponade remains unclear. The aim of this study is to evaluate the “real-world” impact of CF-sensing catheters on cardiac tamponade during AF ablation in an experienced medical center.

**Methods and results:**

This was a retrospective study of consecutive *de novo* AF ablation procedures at Beijing Anzhen Hospital between 2013 and 2016. The ablation procedure was divided into a CF group and a non-CF group. Logistic regression analysis was used to evaluate the association between the use of CF-sensing catheters and the risk of cardiac tamponade. A total of 5313 patients with AF were involved in this study. The incidence of cardiac tamponade in the CF group was significantly higher than that in the non-CF group (1.07% vs. 0.44%, *P* = 0.009). Of the cardiac tamponade cases in the non-CF group, 45.45% were delayed compared with 10% in the CF group (*P* = 0.011). Multivariate logistic regression analysis showed that CF-sensing catheters increased the risk of cardiac tamponade (OR = 2.34, 95% CI = 1.17–4.26, *P* = 0.015). Stratified analysis revealed patients with a smaller left atrium dimension, lower ejection fraction, longer procedure duration, or longer ablation duration had a greater risk of cardiac tamponade during ablation with CF-sensing catheters.

**Conclusions:**

CF-sensing catheters increase the risk of cardiac tamponade during AF ablation.

**Electronic supplementary material:**

The online version of this article (10.1007/s10840-019-00516-z) contains supplementary material, which is available to authorized users.

## Introduction

Cardiac tamponade is a rare but life-threatening complication during catheter ablation for atrial fibrillation (AF) [1, 2]; the incidence of cardiac tamponade is 1.2–1.31% in worldwide surveys [3, 4]. Urgent pericardiocentesis is often performed; occasionally surgical repair is needed. Cardiac tamponade is the leading cause of perioperative death for AF ablation [5]. It is still a challenge in clinical practice to prevent the occurrence of cardiac tamponade during the ablation procedure despite advances in devices and ablation strategies.

In recent years, contact force (CF)–sensing catheters have been widely adopted in AF ablation. They provide the operator with real-time monitoring of contact pressure between the ablation catheter tip and cardiac tissue. Proper catheter pressure enhances ablation efficiency, increases transmural injury, and reduces recovery of electrical conduction between the pulmonary vein and the left atrium, therefore significantly improving the success rate of the procedure [6]. It is well appreciated that ablation with high CF can cause myocardial perforation and increase the possibility of steam pop, resulting in cardiac tamponade [7]; therefore, theoretically monitoring CF during AF ablation would decrease the occurrence of cardiac tamponade. Due to the very low incidence of cardiac tamponade, previous studies were not powered to demonstrate whether or not the application of CF-sensing catheters in clinical practice would reduce the incidence of cardiac tamponade, as they enrolled a small number of patients [8, 9]. In the present study, we attempt to address this issue by analyzing the data from an experienced center with a high volume of AF ablation cases.

## Methods

### Study population

The study was a retrospective investigation of consecutive patients who underwent *de novo* radiofrequency ablation of AF at Beijing Anzhen Hospital from January 2013 to December 2016. Paroxysmal AF (PAF) was defined as sustained AF of less than 7 days. Persistent AF (PerAF) was defined as continuous AF that was sustained beyond 7 days. Patients were excluded if they had combined AF catheter ablation and left atrial appendage closure. This study was approved by the ethics committee of Beijing Anzhen Hospital. Written informed consent was obtained from all patients.

### Periprocedural anticoagulation

An uninterrupted warfarin approach was routinely used in our practice. Low molecular weight heparin (LMWH) was administrated at 1 mg/kg q12h for patients with INR < 2 or without oral anticoagulants. The superior, accepted INR value should be less than 3. Patients with INR > 3 received a reduced dose of warfarin or discontinued warfarin, and the ablation was postponed 1–2 days until the INR was < 3. Patients on novel oral anticoagulants (NOACs) discontinued these 24–48 h before the procedure and were administered LMWH at 1 mg/kg q12h. The last dose of LMWH was given the night before the procedure. During the process, an intravenous heparin bolus of 100 IU/kg was administered after the transseptal puncture, followed by hourly injections of 1000 IU heparin, to maintain the activated clotting time (ACT) at > 300 s. Post-procedure anticoagulants were recommenced 6–8 h later.

### Ablation procedure

After overnight fasting, the procedure was performed with patients under conscious sedation with fentanyl and midazolam. A deflectable decapolar catheter was positioned in the coronary sinus (CS). After a single successful transseptal puncture, a non-CF-sensing (NAVISTAR® THERMOCOOL® Catheter TC, Biosense-Webster, Inc., Diamond Bar, CA, USA) or CF-sensing (ThermoCool® SmartTouch® Catheter ST, Biosense-Webster, Inc.) catheter was advanced to the left atrium for mapping and subsequent ablation that was guided by a three-dimensional electroanatomic mapping system (CARTO, Biosense-Webster, Inc.). Radiofrequency (RF) energy was delivered with a maximum temperature of 43 °C, a power up to 35 W, and a flow rate of 17 ml/min. When ablating inside the CS, the maximum power was decreased to 25 W while the flow rate was increased to 30 ml/min. At each site, the ablation time was no more than 30 s. The optimal force for the CF-sensing catheter during the procedure was about 10–20 g with the alarm cutoff set at 25 g.

Circumferential pulmonary vein ablation (CPVA) was performed for patients with paroxysmal AF with complete electrical isolation of the pulmonary vein (PV) antrum as the endpoint. A fixed approach, the “2C3L” strategy, was adopted for patients with persistent AF [10]. Briefly, after initial CPVA, linear ablation was empirically applied across the mitral isthmus (MI), the left atrial (LA) roof, and the cavo-tricuspid isthmus (CTI). The procedural endpoint of this strategy was the isolation of the PV antrum and complete conduction block across the three ablation lines. Once regular atrial tachycardia occurred during the procedure, activation and entrainment mapping were performed to define the underlying mechanisms and the critical site for ablation. The procedure was performed by one transseptal crossing and single-catheter technique. At the end of the procedure, a circular mapping was introduced into the pulmonary veins to confirm PV isolation. Intracardiac echo was not used during the procedure. Very few cases with a long CTI were ablated with deflectable sheaths. There were nine operators with a yearly AF ablation volume of > 200 cases in this study.

The non-CF-sensing catheter was used in our center from January 2013 to October 2014. The time between November 2014 and March 2015 was a transition period from the non-CF-sensing catheter to the CF-sensing catheter. Since March 2015, all procedures were performed with the CF-sensing catheter.

### Diagnosis and management of cardiac tamponade

The patients’ vital signs were closely monitored during the procedure. Once patients had dyspnea, nausea, vomiting, vertigo, and a drop in systolic blood pressure to < 90 mmHg, cardiac tamponade was suspected. Pericardial effusion was confirmed by a non-pulsating cardiac border and an epicardial halo on fluoroscopy or an echocardiographic evaluation; acute cardiac tamponade was diagnosed. In patients with an unstable hemodynamic state, urgent fluoroscopy-guided pericardiocentesis as per the physician’s discretion was performed by a subxiphoid approach. A pigtail catheter was placed in the pericardial space. Pericardial fluid was drawn out via the pigtail catheter, and retransfusion of the pericardial blood via a femoral venous sheath was performed. Protamine sulfate was administered to reverse heparin and vitamin K_1_ was used to modify supratherapeutic INR if necessary. The drainage catheter was removed in the catheter lab when the hemodynamic status remained stable, and echocardiographic findings confirmed there was no recurrence of pericardial effusion. Once the patients had an unstable hemodynamic state, despite successful pericardiocentesis, urgent surgical repair was required as per the physician’s discretion. Delayed cardiac tamponade was defined as hypotension or cardiogenic shock requiring pericardial drainage due to documented pericardial effusion occurring at least 1 h post-procedurally but attributable to the ablation procedure. The management of delayed cardiac tamponade was similar to that of acute cardiac tamponade, while the retransfusion of the pericardial blood was not recommended to the patients. LMWH was restored 12 h after removing the drainage catheters, then bridging to the resumption of warfarin or NOACs.

### Statistical analysis

Data were analyzed using SAS Version 9.3 (SAS Institute, Cary, NC, USA). Continuous variables were summarized as the means with standard deviations and were compared using *t* tests. Categorical variables were reported as counts with proportions and were compared using chi-square tests or Fisher’s exact tests. The Cochran-Armitage trend test was used to evaluate the time trend of cardiac tamponade based on its rates. The unadjusted and adjusted odds ratios (OR) and 95% confidence intervals (CI) were calculated using Firth’s bias-reduced logistic regression analysis to evaluate the risk of cardiac tamponade associated with the use of the CF-sensing catheter. Covariates used in the multivariate model included age, sex, AF type, comorbid conditions, oral anticoagulant use, left atrium dimension, ejection fraction, procedure duration, and ablation duration; left atrium dimension, ejection fraction, procedure duration, and ablation duration were categorized as dichotomous variables in the model. Sex, AF type, oral anticoagulant use, left atrium dimension, ejection fraction procedure duration, and ablation duration were reported to be likely associated with the occurrence of cardiac tamponade, and stratified analyses were also conducted. A two-sided *P* value < 0.05 was considered significant.

## Results

### Patients’ characteristics

In this study, 5313 patients were enrolled and evaluated. Two thousand five hundred one patients were in the non-CF group, and 2812 patients were in the CF group. The demographic and clinical characteristics of the patients in the two groups are given in Table 1. Compared with the non-CF group, the CF group had more persistent AF, coronary heart disease, heart failure, prior stroke, oral anticoagulant use, enlarged left atrium, reduced ejection fraction, longer procedure duration, and longer ablation duration.

**Table 1:** Demographic and clinical characteristics of patients

	Non-CF group (*n* = 2501)	CF group (*n* = 2812)	*P* value
Male	65.33%	67.25%	0.141
Age (years)	59.57 ± 10.73	60.59 ± 10.78	< 0.001
Paroxysmal AF	64.65%	59.67%	< 0.001
Hypertension	50.94%	52.92%	0.150
Diabetes	14.99%	15.93%	0.346
Coronary heart disease	11.04%	16.15%	< 0.001
Pacemaker and ICD	1.44%	1.67%	0.496
Heart failure	2.92%	5.48%	< 0.001
Prior stroke	4.12%	7.43%	< 0.001
Oral anticoagulants	79.41%	86.74%	< 0.001
INR	1.80 ± 0.49	18.2 ± 0.46	0.568
LA diameter (mm)	25.05 ± 13.47	38.10 ± 10.26	< 0.001
EF (%)	57.06 ± 21.06	60.27 ± 15.26	0.001
Procedure duration (min)	123.40 ± 42.75	126.60 ± 41.76	0.006
Ablation duration (min)	69.09 ± 24.98	70.49 ± 24.27	0.040

### Characteristics of patients with cardiac tamponade

Cardiac tamponade occurred in 41 cases (0.77%), and none of them died. No tamponade occurred during transseptal maneuver or CS catheter placement. Tamponade occurred in one patient during the electroanatomic reconstruction of the left atrium using the non-CF-sensing catheter before ablation. The CF group had a significantly higher incidence of cardiac tamponade compared with the non-CF group (0.44% vs. 1.07%, *P* = 0.009). Table 2 shows the characteristics of patients with cardiac tamponade in the non-CF group and the CF group. In the non-CF group, 45.45% of cardiac tamponade cases were delayed compared with 10% in the CF group (*P* = 0.011). One patient with cardiac tamponade in the CF group required urgent surgery repair while none in the non-CF group.

**Table 2:** Characteristics of patients with cardiac tamponade

	Non-CF group (*n* = 11)	CF group (*n* = 30)	*P* value
Male	63.64%	66.67%	0.281
Age	66.91 ± 7.83	64.20 ± 9.68	0.411
Paroxysmal AF	81.82%	66.67%	0.209
Hypertension	81.82%	63.33%	0.171
Diabetes	27.27%	6.67%	0.096
Coronary heart disease	18.18%	23.33%	0.320
Heart failure	9.09%	13.33%	0.402
Prior stroke	0.00	13.33%	0.271
Oral anticoagulants	81.82%	80.00%	0.342
LA diameter (mm)	38.45 ± 3.42	40.57 ± 5.90	0.273
EF (%)	65.82 ± 6.51	63.43 ± 8.46	0.406
Procedure duration (min)	170.90 ± 84.32	179.20 ± 64.68	0.741
Ablation duration (min)	91.00 ± 49.05	99.23 ± 36.04	0.561
Delayed cardiac tamponade	45.45%	10%	0.011
Pericardiocentesis	81.81%	96.67%	0.106
Pericardial drain (ml)	343.1 ± 129.4	332.9 ± 163.4	0.535
Surgical repair	0	3.33%	1.000

### Time trend of cardiac tamponade

From 2014 to 2016, the annual incidence of cardiac tamponade was 0.27%, 0.56%, 1.03%, and 1.06%; the Cochran-Armitage trend test showed a significant difference (*P* = 0.005). There was no significant difference in the incidence of cardiac tamponade between 2015 and 2016 in the CF group (*P* = 0.989).

### Risk of cardiac tamponade with the CF-sensing catheter

Univariate logistic regression analysis showed that the CF-sensing catheter increased the risk of cardiac tamponade (OR = 2.37, 95% CI = 1.20–4.69, *P* = 0.013). After adjusting for sex, paroxysmal AF, hypertension, diabetes, coronary heart disease, pacemaker, heart failure, prior stroke, oral anticoagulant use, left atrium dimension, ejection fraction, procedure duration, and ablation duration, multivariate logistic regression analysis still showed that the CF-sensing catheter increased the risk of cardiac tamponade (OR = 2.34, 95% CI = 1.17–4.26, *P* = 0.015; Table 3). Then, stratified analyses were conducted based on sex, oral anticoagulant use, left atrium dimension, ejection fraction, procedure duration, and ablation duration, which were considered to influence the occurrence of cardiac tamponade. Female sex and oral anticoagulant use did not increase the risk of cardiac tamponade with non-CF-sensing catheter ablation, while CF-sensing catheter ablation significantly increased the risk of cardiac tamponade in individuals with a smaller left atrium dimension, lower ejection fraction, longer procedure duration, or longer ablation duration (Table 4).

**Table 3:** Unadjusted and adjusted OR (95% CI) for cardiac tamponade with the CF-sensing catheter

	Cardiac tamponade (*n*, %)	OR (95% CI)	*P*	OR (95% CI)	*P*
Non-CF group	11 (0.44)	1.00	0.013	1.00	0.015
CF group	30 (1.07)	2.37 (1.20, 4.69)		2.34 (1.17, 4.26)

**Table 4:** Stratified analyses of adjusted OR (95% CI) for cardiac tamponade with the CF-sensing catheter

	Cardiac tamponade (*n*, %)	OR (95% CI)	*P*
Male			
Non-CF group	7 (0.43)	1.00	0.080
CF group	20 (1.06)	2.00 (0.92, 4.36)	
Female			
Non-CF group	4 (0.46)	1.00	0.084
CF group	10 (1.09)	2.36 (0.89, 6.21)	
PAF			
Non-CF group	9 (0.56)	1.00	0.062
CF group	20 (1.19)	1.98 (0.97, 4.05)	
PerAF			
Non-CF group	2 (0.23)	1.00	0.101
CF group	10 (0.88)	2.67 (0.83, 8.61)	
Anticoagulant use			
Non-CF group	2 (0.39)	1.00	0.059
CF group	6 (1.61)	1.97 (0.97, 3.99)	
No anticoagulant use			
Non-CF group	9 (0.45)	1.00	0.034
CF group	24 (0.98)	3.78 (1.10, 12.95)	
Left atrium dimension < 40 mm			
Non-CF group	7 (0.44)	1.00	0.024
CF group	16 (1.11)	2.47 (1.13, 5.43)	
Left atrium dimension ≥ 40 mm			
Non-CF group	4 (0.44)	1.00	0.152
CF group	14 (1.03)	2.00 (0.78, 5.15)	
Ejection fraction < 64%			
Non-CF group	3 (0.21)	1.00	0.002
CF group	16 (1.13)	5.04 (1.80, 14.13)	
Ejection fraction ≥ 64%			
Non-CF group	8 (0.73)	1.00	0.550
CF group	14 (1.0)	1.27 (0.58, 2.78)	
Procedure duration < 120 min			
Non-CF group	2 (0.19)	1.00	0.196
CF group	5 (0.46)	2.15 (0.67, 6.87)	
Procedure duration ≥ 120 min			
Non-CF group	9 (0.61)	1.00	0.030
CF group	25 (1.44)	2.18 (1.08, 4.43)	
Ablation duration < 67 min			
Non-CF group	4 (0.31)	1.00	0.229
CF group	7 (0.52)	1.83 (0.68, 4.91)	
Ablation duration ≥ 67 min			
Non-CF group	7 (0.59)	1.00	0.025
CF group	23 (1.56)	2.42 (1.12, 5.25)	

## Discussion

The present study demonstrated that the CF-sensing catheter did not decrease the incidence of cardiac tamponade in AF ablation. Multivariate regression analysis showed that the CF-sensing catheter increases the risk of cardiac tamponade during the ablation procedure.

There is an association between the occurrence of cardiac tamponade and the experience of hospitals and operators. Deshmukh et al. analyzed 93,391 cases of atrial fibrillation catheter ablation in the USA between 2000 and 2010; the annual operation volume of hospitals (< 50 cases) and the surgery year (< 25 cases) were associated with severe complications of AF catheter ablation [11]. The study by Bollmann et al. also confirmed the volume-outcome relationship in AF ablation [12]. In the present study, the yearly AF ablation volume of the operator was > 200 cases, and the incidence of cardiac tamponade in the non-CF group was 0.44%, which was much lower than that in a global survey conducted by Cappato et al. in the era of non-CF-sensing catheters [3, 4]. Thus, our data may be representative of experienced AF ablation centers in the real world.

Animal studies and clinical studies have clearly demonstrated that the contact force between the catheter tip and cardiac tissue is related not only with the extent of lesions but also with the occurrence of some serious complications, such as cardiac perforation. Perna et al. reported that a contact force > 77 g could directly produce a perforation of the atrial wall in a swine model and ablation reduced the perforating force by 23% [13]. Di Biase et al. systemically explored the relationship between catheter forces, steam “popping,” and crater formation using an irrigated catheter. They found that using a pressure of 20–30 g, a power of 45 W, and an ablation time of 40 s caused “popping” and crater formation in 33.3% of ablation sites, while none of the sites developed these conditions when 10 g of pressure was used [14]. The study by Ikeda et al. obtained similar data [7]. In another investigation of AF patients, Makimoto et al. demonstrated that CF was closely associated with crater formation at the tip of the catheter and suggested that CF should be controlled to < 25 g to reduce serious complications [15].

There is growing evidence that the utilization of the CF-sensing catheter during AF ablation significantly improves AF ablation success rate as compared with the traditional non-CF-sensing catheter; thus, the CF-sensing catheter has been widely and quickly adopted in many medical centers, as well as in our center. The occurrence of cardiac tamponade is associated with CF between the catheter tip and atrial tissue; it is enthusiastically believed that the application of CF-sensing catheters would lower the incidence of cardiac tamponade in the AF ablation procedure. Initial studies have demonstrated that CF-sensing catheters did not increase perioperative complications compared with non-CF-sensing catheters. Since the incidence of cardiac tamponade is very low, those studies are not powered to demonstrate the safety of CF-sensing catheters in preventing rare complications during the procedure. For example, in the TOCCASTAR study, one case of cardiac tamponade occurred in each group [9], while all four cases of cardiac tamponade in the SMART-AF study happened in the CF-sensing catheter group [8]. In the present study, the CF group had a higher incidence of cardiac tamponade compared with the non-CF group. The transition period from the non-CF-sensing catheter to the CF-sensing catheter is during October 2014 to February 2015; we observed a significant increase in cardiac tamponade during the study period. There was no significant difference in the occurrence of cardiac tamponade between 2015 and 2016; therefore, the impact of the learning curve of the CF-sensing catheter was excluded [16]. The patients in the CF group had more comorbid conditions, such as coronary heart disease, heart failure, and prior stroke; moreover, the procedure duration and the ablation duration were longer in the CF group than in the non-CF group. Subsequently, multivariate regression analysis showed that the CF-sensing catheter still increased the risk of cardiac tamponade. Yang et al. analyzed 1475 cases of AF ablation in Johns Hopkins Hospital over 12 years; the incidence of cardiac tamponade was 1.1%, and the CF-sensing catheter did not reduce the occurrence of cardiac tamponade [17]. Hamaya et al. reported that the incidence of cardiac tamponade in the CF group was higher than that in the non-CF group (2.1% vs. 1.3%) from 1815 patients with paroxysmal AF undergoing catheter ablation, while there was no statistical difference [18]. A prospective study by Akca et al. found that cardiac tamponade only occurred in the non-CF group (3.3%) and none in the CF group [19], which was not in agreement with our study. The incidence of cardiac tamponade in Akca et al.’s study was much higher than that in worldwide surveys, and the annual case of AF ablation was less than 100, so we speculate a discrepancy in AF ablation cases between the two studies.

There is an imbalance between the baseline characteristics of the non-CF group and the CF group. The cases in the CF group had more PerAF, a larger left atrium, and a higher rate of use of oral anticoagulants. Apparently linear ablation in PerAF, a thinner left atrial wall, a higher rate of anticoagulant use, a longer procedure duration, and a longer ablation duration in the CF group would increase the propensity for the occurrence of cardiac tamponade. These confounding factors would be a potential bias. Next, we performed the stratified analyses in the subgroups. Cardiac tamponade was compatible between the non-CF group and the CF group in the subgroup of PerAF; in addition, oral anticoagulant use did not increase cardiac tamponade, which is consistent with previous observations [20]. Interestingly we did observe that there was more cardiac tamponade in the CF group in the subgroup of patients with a smaller left atrium, longer procedure duration, or longer ablation duration. Since all cardiac tamponade cases happened after the transseptal puncture, we speculated that it is likely that the increased number of cardiac tamponade events in the CF group is associated with the catheter manipulation.

It is interesting to note that 45.45% of cardiac tamponade events in the non-CF group were delayed; on the contrary, only 10% were observed in the CF group. The global survey conducted in the era of non-CF-sensing catheters showed that hospitals with an annual volume of > 299 cases are more prone to delayed cardiac tamponade [21], which is consistent with our observation. One case with cardiac tamponade in the CF group required urgent surgical repair, but none in the non-CF group. Therefore, our data is likely to indicate that cardiac tamponade induced by the CF-sensing catheter is more urgent compared with that by the non-CF-sensing catheter.

The optimal CFs were targeted between 10 and 20 g with a cutoff CF of 25 g in the present study. Therefore, excessive CF-induced cardiac tamponade was extensively excluded, while we did not observe an improvement in cardiac tamponade after application of the CF-sensing catheter. We propose the following interpretations: (1) Although CF is a critical factor for cardiac tamponade, the occurrence of cardiac tamponade is the result of an extensive interaction among CF, ablation power, time, temperature, and impedance; therefore, all the above parameters should be carefully monitored during the ablation procedure [22]. (2) In the stratified analyses, the patients with a smaller left atrium dimension had a greater risk of cardiac tamponade during the ablation procedure with the CF-sensing catheter. Given the facts that the tip of the CF-sensing catheter is stiffer compared to that of the non-CF-sensing catheter and CF measurements are sampled every 50 ms and calibrated to produce a contact force reading that is averaged over 1 s [8], cardiac tamponade is likely to be associated with the manipulation of the CF-sensing catheter, and instant CF may be excessive to cause cardiac perforation. (3) Operators using the CF-sensing technology to target optimal CF may ablate longer and with greater force than those using the non-CF-sensing catheter [23]; increased energy delivery with CF-sensing catheter–guided ablation might increase the risk of cardiac tamponade, which seems to be consistent with the fact that few delayed cardiac tamponade cases occurred in the CF group.

### Study limitation

The most important limitation of our study was its retrospective and observational design. There was no data on audible steam pop, the possible location of the perforation, and the exact CF during the procedure. The imbalance between baseline characteristics of the two groups was another limitation. The propensity-matched cases in the two groups may eliminate the bias of these confounding factors. This study may be not powered to address the issue because of the low incidence of cardiac tamponade despite the large sample size in the present study. Additionally, the present study came from an experienced center and operators, so it is still questionable whether CF-sensing catheters would increase cardiac tamponade in medical centers with a low volume of cases.

## Conclusion

Cardiac tamponade is a rare but life-threatening complication during catheter ablation for AF. In this retrospective study from an experienced center, unexpectedly, our data show that the CF-sensing catheter increases the risk of cardiac tamponade during AF ablation. It highlights that CF monitoring cannot be guaranteed to avoid cardiac tamponade. Caution should be taken in using the CF-sensing catheter for AF ablation, and an optimized ablation strategy for CF-sensing catheters is needed to minimize the risk of cardiac tamponade.

## Electronic supplementary material

ESM 1 (DOCX 50 kb)
