# Supplementary material for: Association between the use of contact force-sensing catheters and cardiac tamponade in atrial fibrillation ablation
Source: J Interv Card Electrophysiol. 2019 Feb 2;55(2):137–43. doi: 10.1007/s10840-019-00516-z (PMC6660577; doi:10.1007/s10840-019-00516-z)
Supplement: Supplementary file 1 — (DOCX 50 kb) [file 10840_2019_516_MOESM1_ESM.docx]

Supplemental table: Characteristics of patients with cardiac tamponade

| Case | Gender / Age | Type of AF | Type of catheter | Oral Anticoagulants | INR | Heparin dose during procedure (U) | ACT before tamponade (s) | Time of delayed tamponade after procedure (min) | Pericardiocentesis | Pericardial drain (ml) | Surgical repair |
| --- | --- | --- | --- | --- | --- | --- | --- | --- | --- | --- | --- |
| 1 | M/61 | PAF | Non-CF | [Warfarin](javascript:;) | 2.1 | 6000 | >300 | 70 | Yes | 430 | No |
| 2 | F/79 | PAF | CF | Dabigatran | - | 6000 | >300 | 70 | Yes | 180 | No |
| 3 | F/76 | PAF | CF | [-](javascript:;) | - | 6000 | >300 | - | Yes | 490 | No |
| 4 | F/65 | PerAF | Non-CF | [Warfarin](javascript:;) | 2.8 | 5000 | >300 | - | Yes | 310 | No |
| 5 | F/63 | PAF | CF | [-](javascript:;) | - | 7000 | >300 | - | Yes | 230 | No |
| 6 | M/46 | PAF | CF | [-](javascript:;) | - | 6000 | 494 | 180 | Yes | 350 | No |
| 7 | M/56 | PAF | CF | Dabigatran | - | 6000 | 348 | - | Yes | 330 | No |
| 8 | M/65 | PAF | Non-CF | [Warfarin](javascript:;) | 1.6 | 5000 | >300 | - | Yes | 230 | No |
| 9 | F/72 | PAF | Non-CF | [-](javascript:;) | - | 6000 | >300 | 1080 | Yes | 200 | No |
| 10 | F/74 | PAF | CF | Dabigatran | - | 6000 | >300 | - | Yes | 160 | No |
| 11 | F/76 | PAF | CF | Rivaroxaban | - | 6000 | >300 | - | Yes | 580 | No |
| 12 | F/68 | PAF | CF | Dabigatran | - | 6000 | >300 | - | Yes | 240 | No |
| 13 | M/66 | PAF | Non-CF | [-](javascript:;) | - | 6000 | >300 | - | No | - | N0 |
| 14 | M/66 | PAF | CF | Rivaroxaban | - | 6000 | >300 | - | Yes | 240 | No |
| 15 | M/67 | PAF | Non-CF | [Warfarin](javascript:;) | 1.2 | 6000 | >300 | - | No | - | No |
| 16 | M/48 | PAF | CF | [Warfarin](javascript:;) | 1.9 | 6000 | >300 | - | Yes | 700 | No |
| 17 | M/60 | PAF | CF | [Warfarin](javascript:;) | 1.3 | 6000 | >300 | - | Yes | 200 | No |
| 18 | M/64 | PAF | CF | Rivaroxaban | - | 6000 | >300 | - | Yes | 230 | No |
| 19 | M/70 | PerAF | CF | [Warfarin](javascript:;) | 1.8 | 7000 | 835 | - | Yes | 470 | No |
| 20 | M/55 | PerAF | CF | [-](javascript:;) | - | 6000 | >300 | - | Yes | 180 | No |
| 21 | M/56 | PerAF | CF | Dabigatran | - | 6000 | >300 | 70 | No | - | No |
| 22 | M/60 | PAF | Non-CF | [Warfarin](javascript:;) | 0.85 | 6000 | >300 | 85 | Yes | 450 | No |
| 23 | M/61 | PAF | CF | Rivaroxaban | - | 6000 | >300 | - | Yes | 400 | No |
| 24 | M/73 | PAF | Non-CF | [Warfarin](javascript:;) | 2.6 | 6000 | >300 | 110 | Yes | 600 | No |
| 25 | F/65 | PAF | CF | Dabigatran | - | 5000 | >300 | - | Yes | 550 | No |
| 26 | F/64 | PAF | CF | Dabigatran | - | 6000 | >300 | - | Yes | 300 | No |
| 27 | M/58 | PerAF | CF | Dabigatran | - | 6000 | >300 | - | Yes | 670 | No |
| 28 | M/72 | PerAF | CF | Rivaroxaban | - | 6000 | >300 | - | Yes | 190 | No |
| 29 | M/53 | PerAF | CF | Rivaroxaban | - | 6000 | 650 | - | Yes | 200 | No |
| 30 | F/75 | PerAF | Non-CF | [-](javascript:;) | - | 5000 | >300 | - | Yes | 240 | No |
| 31 | M/86 | PAF | CF | [-](javascript:;) | - | 6000 | >300 | - | Yes | 200 | No |
| 32 | M/77 | PerAF | CF | Rivaroxaban | - | 7000 | >300 | - | Yes | 380 | No |
| 33 | F/60 | PAF | CF | [-](javascript:;) | - | 6000 | >300 | - | Yes | 330 | No |
| 34 | M/61 | PAF | CF | Rivaroxaban | - | 6000 | >300 | - | Yes | 250 | No |
| 35 | F/80 | PAF | Non-CF | [Warfarin](javascript:;) | 2.6 | 5000 | >300 | 70 | Yes | 280 | No |
| 36 | M/75 | PerAF | CF | Rivaroxaban | - | 6000 | >300 | - | Yes | 300 | No |
| 37 | M/60 | PerAF | CF | Rivaroxaban | - | 7000 | 540 | - | Yes | 600 | No |
| 38 | M/63 | PerAF | CF | Dabigatran | - | 6000 | >300 | - | Yes | - | Yes |
| 39 | F/64 | PAF | CF | [-](javascript:;) | - | 6000 | 827 | - | Yes | 210 | No |
| 40 | M/50 | PAF | CF | [Warfarin](javascript:;) | 1.93 | 6000 | 612 | - | Yes | 160 | No |
| 41 | M/52 | PAF | Non-CF | [Warfarin](javascript:;) | 1.2 | 6000 | 244 | - | Yes | 348 | No |
